# Hair Growth Activity of Three Plants of the Polynesian Cosmetopoeia and Their Regulatory Effect on Dermal Papilla Cells

**DOI:** 10.3390/molecules25194360

**Published:** 2020-09-23

**Authors:** Kristelle Hughes, Raimana Ho, Stéphane Greff, Edith Filaire, Edwige Ranouille, Claire Chazaud, Gaëtan Herbette, Jean-François Butaud, Jean-Yves Berthon, Phila Raharivelomanana

**Affiliations:** 1 IFREMER, ILM, IRD, EIO UMR 241, Université Polynésie Française, BP 6570, F-98702 Faaa, Tahiti, French Polynesia; kristelle.hughes@doctorant.upf.pf (K.H.); raimana.ho@upf.pf (R.H.); 2 Institut Méditerranéen de Biodiversité et d’Ecologie Marine et Continentale (IMBE), UMR 7263 CNRS, IRD, Aix Marseille Université, Avignon Université, Station Marine d’Endoume, rue de la Batterie des Lions, 13007 Marseille, France; stephane.greff@imbe.fr; 3 Greentech SA, Biopôle Clermont-Limagne, 63360 Saint-Beauzire, France; edithfilaire@greentech.fr (E.F.); developpement@greentech.fr (E.R.); jeanyvesberthon@greentech.fr (J.-Y.B.); 4 UMR 1019 INRA-UcA, UNH (Human Nutrition Unity), ECREIN Team, Université Clermont Auvergne, 63000 Clermont-Ferrand, France; 5 GReD Institute, Université Clermont Auvergne, CNRS, Inserm, Faculté de Médecine, CRBC, 63000 Clermont-Ferrand, France; claire.chazaud@uca.fr; 6 Aix Marseille Univ, CNRS, Centrale Marseille, FSCM, Spectropole, Service 511, Campus Saint-Jérome, 13397 Marseille CEDEX 20, France; gaetan.herbette@univ-amu.fr; 7 Consultant in Forestry and Polynesian Botany, BP 52832, 98716 Pirae, Tahiti, French Polynesia; jfbutaud@hotmail.com

**Keywords:** Polynesian plants, ethnobotany, cosmetopoeia, dermal papilla, hair growth, Wnt

## Abstract

Hair loss is becoming increasingly prevalent as dietary and living habits change. The search for natural products to limit hair loss has led to tapping into traditional cosmetic knowledge. We studied three plants of the Polynesian cosmetopoeia, *Bidens pilosa*, *Calophyllum inophyllum* and *Fagraea berteroana*, to determine their ability to promote hair growth. Their chemical content was characterized by liquid chromatography coupled to mass spectrometry (LC-MS). Their proliferative activity on dermal papilla cells (DPCs) was assessed via MTT assay and molecular targets were evaluated by RT-qPCR analysis of seven factors involved in the modulation of the hair cycle, *CCND1*, *LEF1*, *DKK1*, *WNT5A PPARD*, *TGFΒ1*, *PPARD* and *RSPO2*. Our results show that our extracts significantly increased proliferation of dermal papilla cells. Furthermore, LC-MS/MS analysis revealed a diversity of molecules, flavonoids, iridoids and organic acids, some known for hair-inducing properties. Finally, specific extracts and fractions of all three plants either upregulated *CCND1*, *LEF1* and *PPARD* involved in stimulating hair follicle proliferation and/or lowered the gene expression levels of hair growth inhibiting factors, *DKK1* and *TGFB1*. Our findings suggest that extracts from *B. pilosa, C. inophyllum* and *F. berteroana* are interesting candidates to stimulate hair growth.

## 1. Introduction

Cosmetopoeia refers to the use of plants or minerals for the care and embellishment of the body and its attributes [1]. In French Polynesia, different types of ointments, plant mixtures, are used traditionally to maintain good hygiene and beautify the skin and hair of both men and women. Previous studies of plants’ medicinal and cosmetic potential in the region have revealed a variety of uses for hair growth, skin hydration, wound healing and toiletries [2,3,4]. A former selection of eleven plants of the Polynesian cosmetopoeia was achieved by studying diverse literary sources concerning the ethno-uses of plants present in French Polynesia and their known phytochemistry data within bioactivities related to body care [5]. The obtained phytoextracts were tested for their anti-inflammatory and antioxidant activities and seven interesting extracts (from four plants) for body and hair care were revealed, when both literature data and the tested bioassays were taken into account [5].

Among the plants formerly investigated, only three plants were retained for our present study, *Bidens pilosa* L., *Calophyllum inophyllum* L. and *Fagraea berteroana* A.Gray ex Benth., because the plant part studied for the fourth one, the bark of *Tephrosia purpurea* (L.) Pers. var. *purpurea*, could prove difficult to be collected in bulk amount.

The three plants are used for a variety of traditional cosmetic uses in the Indo-Pacific cosmetopoeia. The fruits of *F. berteroana* were crushed and applied on cadavers’ hair during embalming in the Marquesas Islands to prevent hair loss [6]. Its fragrant flowers are used to perfume coconut oil and obtain scented monoi [7]. The nut oil obtained from *C. inophyllum* is used in Tahiti as hair oil to promote healthy and long hair whether on its own or mixed with coconut oil [8,9], while in Tonga and Samoa, the flowers of the tree are used to perfume coconut oil for scalp care [10]. The different parts of this plant, nut oil, leaves and bark, are nevertheless mainly known in the Polynesian cosmetopoeia for wound healing [3,4] and treating skin problems (e.g., eczema, rashes, boils, pimples, skin inflammation) [3,4,10,11,12,13,14,15,16]. Lastly, the crushed or whole leaves of *B. pilosa* were used for treatments to dress wounds and boils and to ease eye inflammation [8,10,17]. The whole plant was used to dress cuts in the Marquesas and Rapa [10].

As presented above, two plants are used to treat both hair and skin, while the other has ethnopharmacological uses for skin problems. Hair care is deeply rooted in the Polynesian traditions, yet few to no studies on hair-related activities of plants of the Polynesian cosmetopoeia have been conducted. This led to our interest in developing plant extracts and propose them as hair growth ingredients.

Currently, minoxidil is one of the few FDA-approved drugs administered to patients to induce or stimulate hair growth. It is prescribed as a topical cream or foam to limit hair loss caused by androgenetic alopecia in both men and women [18,19]. Although its exact mode of action is still being investigated, several studies have shed light on how it exerts its hair growth activity. Among several reported activities, it is known to stimulate the proliferation of the dermal papilla (DP) in a dose-dependent manner [20]. The DP are specialized mesenchymal cells at the base of the hair follicle. They signal progenitor epithelial and stem cells to differentiate, migrate and form the hair shaft [21,22,23]. This action is mediated via the Wnt pathway mainly. The canonical Wnt pathway, also known as the Wnt/β-catenin pathway, is highly expressed during elongation of the hair and leads to a greater expression of nuclear β-catenin protein and downstream target genes of this pathway [24]. The involvement of the non-canonical Wnt pathways in hair growth is poorly understood, although studies have shown that Wnt5α attenuates the Wnt/β-catenin pathway [25,26], and another demonstrates that it could activate it in the presence of a specific receptor (Frizzled 4) [27].

The hair cycle comprises three main states, the anagen or growth phase during which hair shaft elongation is induced, the catagen phase, also known as the apoptotic or involution phase, during which the epithelial cell population regresses drastically and finally the telogen or resting phase that concludes the cycle by allowing shedding of the hair follicle and preparation for entry into a new anagen phase [28,29]. Researchers that study hair growth focus on two key transition phases, anagen to catagen and telogen to anagen [28,30,31], because potent hair growth inducers should deter entry into catagen and/or promote transitioning to a new anagen phase.

In this study, we evaluated the hair growth-promoting effect of three plant extracts of the Polynesian cosmetopoeia, having traditional uses related to hair or skin treatment. We focused on both the potential bioactivities and the chemical composition of the phytoextracts of interest. Extracts were fractioned, and bioassays were performed to determine their anti-inflammatory activity via the 5-lipoxygenase inhibition assay and their antioxidant effect using the ferric reducing antioxidant power (FRAP) method. Furthermore, the resulting extracts were chemically assessed through colorimetric assays and Liquid chromatography coupled to high-resolution mass spectrometry (LC-MS) analysis. Once the most active and promising fractions were chosen, a more in-depth study of their effect on the expression of factors involved in the hair cycle was conducted by following their gene regulation by RT-qPCR in DPCs. Our results enable us to gain better insight into how the extracts exert their hair growth stimulatingactivity regarding potential active compounds and their related hair cycle targets, at the molecular level. So, herein, due to the preliminary results and ethnobotanical data on these plants, the present work focuses on the hypothesis that the studied extracts should induce proliferation of DPCs and their signaling, via regulation of key factors of the Wnt/β catenin pathway.

## 2. Results

### 2.1. Chemical Characterization of Plant Extracts

#### 2.1.1. Colorimetric Assays

**Figure 1:**
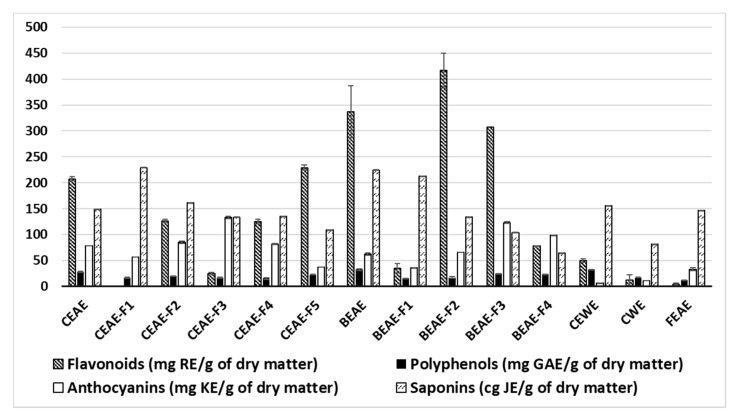
Flavonoid, polyphenol, anthocyanin and saponin levels of the different extracts determined by colorimetric methods. *C. inophyllum* aqueous extract = CWE, ethyl acetate extract = CEAE. *B. pilosa* ethyl acetate extract = BEAE and *F. berteroana* ethyl acetate extract = FEAE. CEAE-F1 to CEAE-F5 = fractions obtained from CEAE and BEAE-F1 to BEAE-F4 = fractions obtained from BEAE. RE = rutin equivalents, GAE = gallic acid equivalents, KE = kuromanin equivalents and JE = jujuboside equivalent.

The polyphenol, flavonoid, anthocyanin and saponin contents of the extracts were assessed by colorimetric methods, as shown in Figure 1. According to our results, *Bidens pilosa* shows the highest flavonoid content. Indeed, the *B. pilosa* ethyl acetate extract (BEAE) of the plant has 337 mg g^−1^ rutin equivalents. Two fractions resulting from BEAE named BEAE-F2 and BEAE-F3 also reveal an interesting flavonoid content above 300 mg g^−1^ rutin equivalents. Similarly, *C. inophyllum* ethyl acetate extract (CEAE) is at 208 mg g^−1^ rutin equivalents and three of its resulting fractions CEAE-F2, CEAE-F4 and CEAE-F5 are at 127, 125 and 230 mg g^−1^ rutin equivalents, respectively. Both the *C. inophyllum* ethanol/water extract (CEWE) and aqueous extract (CWE), as well as the ethyl acetate extract of *F. berteroana* (FEAE), have values below 60 mg g^−1^ rutin equivalents. The polyphenol content of the extracts and fractions shows that CEAE, BEAE and CEWE have the highest values. The saponin content was of the same order of magnitude in all the extracts, ranging between 65 and 229 cg g^−1^ jujuboside equivalent. Nevertheless, the least polar fractions such has CEAE-F1, CEAE-F2, BEAE-F1 show a higher saponin content than the more polar fractions and extracts.

Overall, CEAE, CEAE-F2, CEAE-F3, CEAE-F4, CEAE-F5, BEAE, BEAE-F2, BEAE-F3 and BEAE-F4 show a greater chemical variety than FEAE and CWE. The fractions resulting from FEAE (data not shown) revealed low concentrations of all four families determined.

#### 2.1.2. Characterization and Structural Determination of Compounds by Ultra Performance Liquid Chromatography UPLC-MS/MS

The UPLC-MS/MS analysis of the different extracts in negative ionization mode revealed a series of compounds. Eighteen compounds were tentatively identified by comparing their MS^2^ data with entries in repositories such as MassBank [32] and MassBank of North America (MoNA) [33] and with the literature. The compilation of the *m*/*z*, molecular formulas and potential identification are presented in Table 1. Additional information on the compounds corresponding to chromatogram peaks is supplied in Table S1.

**Figure 2:**
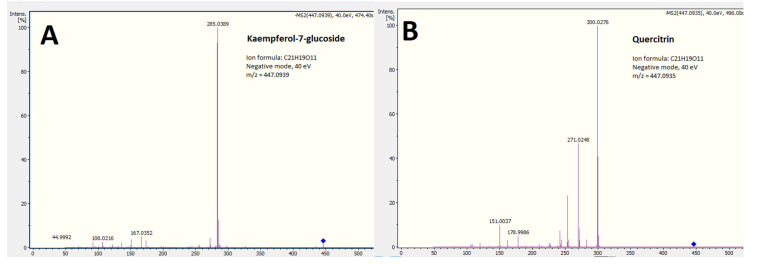
UPLC-MS/MS fragmentation spectra of putative kaempferol-7-glucoside (**A**) and quercitrin (**B**) at 40 eV in negative ionization mode, *m*/*z* = 447.093 present in extracts of all three plant species. The blue diamond symbol represents the parent ion mass before MS^2^ fractioning.

Among the compounds common to all or most of our extracts and fractions were flavonoids, mainly *O*-flavone glycosides (Figure 2) and phenolic acids. Sulfur-containing flavones were tentatively identified in the most polar extracts of *C. inophyllum*, CEWE and CWE, while flavanols were found in all three *C. inophyllum* extracts. An iridoid was also identified, but only in *F. berteroana* extracts.

##### *O*-glycosyl Flavonoids

Flavonoids are polyphenolic compounds commonly found in plants and herbs. Their basic structure is a C6-C3-C6 pattern composed of two benzene rings surrounding a pyran ring. Further hydroxyl and methyl groups complexify the aglycone structure. Here, we first tentatively identified several *O*-glycosyl flavones and flavonols from our different extracts consisting of flavone aglycones with a sugar group attached to one of their hydroxyl groups.

The MS^2^ spectrum of compound 7 (*m*/*z* 433.1740) shows a main fragment at 268.037 (100). This fragment seems to correspond to the flavone aglycone with a loss of 2H, the fragment for the aglycone being at 271.063 (50.9). Indeed, [M-H-C_6_H_10_O_5_] = 271.1212 Da. The fragment at 271.063 was attributed to naringenin. Thus, the ion was identified as naringenin-glucoside. It was further verified by fragment 151.004 (52.2) which is [(M-H-120)-271.063], suggesting an *O*-glucoside. The glycoside loss is observed by 433.1740 − 271.063 = 162.111. Thus, this peak was tentatively identified as prunin.

**Figure 3:**
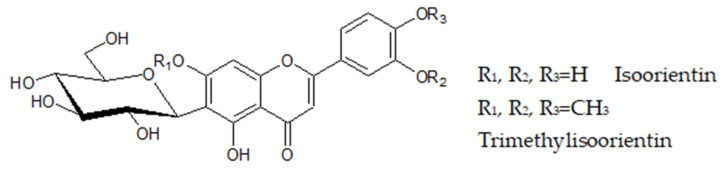
Isoorientin and trimethylisoorientin.

The MS spectrum of compound 14 reveals a peak at 489.1414 [M − H]^−^ which was hypothesized to be trimethylisoorientin (trimethyl luteolin-6-glucoside). Indeed, the MS/MS spectra reveal the loss of a hexoside, 162 Da, as the most dominant fragment is at 327.088 ([M − H]-hexoside) = 327.088. This fragment is a typical MS^2^ fragment of isoorientin (PR040130 in MoNA). Furthermore, isoorientin has a [M−H] mass of 447.08. Indeed, 489.1414 − 447 = 42. These are the three CH_2_ groups believed to be on three hydroxyl (OH) groups of the molecule, as seen in Figure 3.

### 2.2. Biological Assays

#### 2.2.1. Antioxidant (FRAP) and Anti-inflammatory (5-LOX) Activities of Extracts In Vitro

Tests were performed on the extracts to assess their antioxidant and anti-inflammatory potential (Table 2). A dried green tea extract concentrated to 90% polyphenols (900017 internal reference) was taken as a reference for the FRAP assay and nordihydroguaiaretic acid (NDGA) for 5-LOX. The most active extracts corresponded to the most polar fractions of *C. inophyllum* (CEAE-F5) and *B. pilosa* (BEAE-F4) that both demonstrated a Trolox equivalent value above 1000. This reveals that they have a significant antioxidant activity.

Overall, IC50 values of *C. inophyllum* organic fractions (CEAE-F2 to CEAE-F5) showed the best anti-inflammatory activities among all the extracts, ranging from 20 to 28 µg mL^−1^. The aqueous *Calophyllum* extract (CWE) also demonstrated an interesting anti-inflammatory activity with an IC_50_ of 55 μg mL^−1^. Conversely, *F. berteroana* fractions (FEAE-F0 to FEAE-F3) are among the least potent anti-inflammatory extracts.

The selection of the extracts further tested on the DPCs was done by evaluating if the extracts fared well either in bioactivity, or chemical composition, or both. This led to retaining CEWE, CEAE, CEAE-F3, CEAE-F4, CEAE-F5, BEAE, BEAE-F3, BEAE-F4, FEAE, FEAE-F0, FEAE-F1, FEAE-F2 and FEAE-F3. The latter five were maintained to study the full potential of *F. berteroana* further, as it is the least studied plant of the three according to a literature search.

#### 2.2.2. Proliferative Effect of Extracts and Fractions on Dermal Papilla Cells after 24 h and 48 h Treatment

To better determine the hair proliferative effect of the extracts, minoxidil was taken as a reference. It also led to choosing two treatment periods for our study. Cell proliferation was studied for 24 h and 48 h. The treatment period of 24 h was chosen because several previous studies have shown that after 24 h, minoxidil already exerts proliferative properties on hair follicles and DP cells [35,36]. Nevertheless, a 48-h long treatment gave a perspective on the efficiency of our extracts over a longer period. Three concentrations of minoxidil were tested on DPCs proliferation, 0.13, 1.25 and 12.5 μg mL^−1^, corresponding to 0.6, 6 and 60 μM, respectively. Indeed, a study showed that at micromolar concentrations, minoxidil induces cell proliferation of keratinocytes, whereas at millimolar concentrations, it leads to cell death [37]. The other extracts were tested at seven concentrations: 0.10, 1.56, 3.13, 6.25, 12.5, 25 and 50 μg mL^−1^. The control corresponds to the solvent control 100% DMSO at concentrations equivalent to that of the treatments with extracts between 0.001% to 0.5%. The tested DMSO dilutions did not influence cell growth (data not shown) when compared to cells that were solely grown in cell medium with no added substance. The statistical significance presented below for cell proliferation and RT-qPCR analysis was done using an unpaired Student’s *t*-test or Welch’s *t*-test if variances were equal or unequal, respectively. Detailed p-values for each value obtained are shown in Table S2. A *p*-value ≤ 0.05 was considered significant.

**Figure 4:**
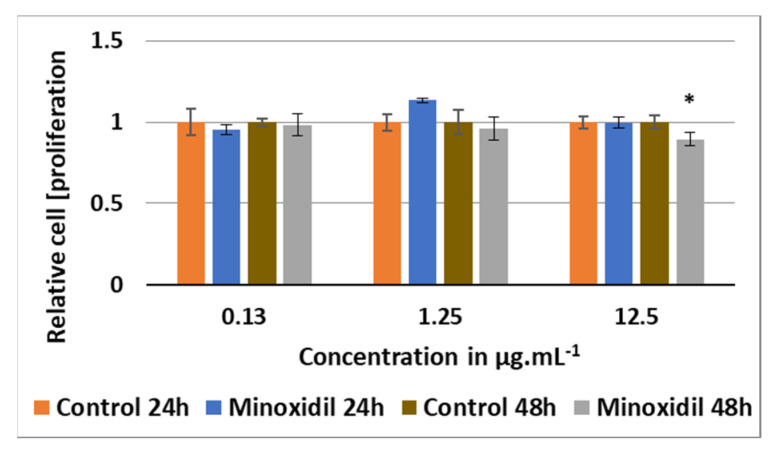
Cell-proliferating effect of minoxidil after 24 h and 48 h of treatment of dermal papilla cells with concentrations 0.13, 1.25 and 12.5 μg mL^−1^ compared the control. Significance is determined by * *p* < 0.05, using a Student’s *t*-test.

**Figure 5:**
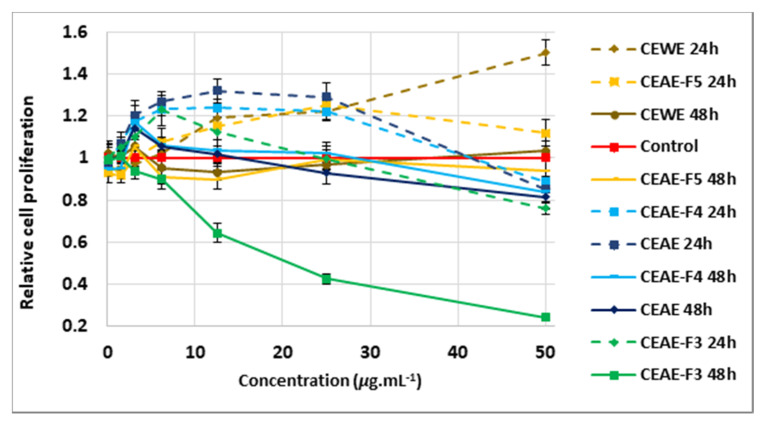
Cell-proliferating effect of *C. inophyllum* extracts, CEWE and CEAE, and resulting fractions CEAE-F3, CEAE-F4 and CEAE-F5 after 24 h and 48 h of treatment of dermal papilla cells at concentrations 0.1 to 50 μg mL^−1^ normalized and compared to the control. Error bars represent SEM.

**Figure 6:**
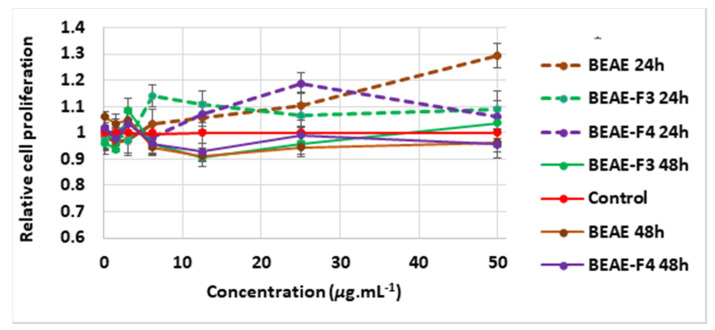
Cell proliferating effect of *B. pilosa* ethyl acetate extract (BEAE) and resulting fractions BEAE-F3 and BEAE-F4 after 24 h and 48 h of treatment of dermal papilla cells with concentrations 0.1 to 50 μg mL^−1^ normalized and compared the control. Error bars represent SEM.

**Figure 7:**
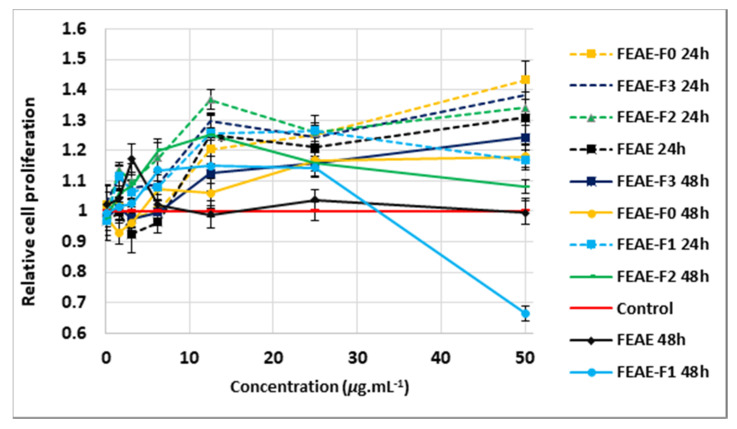
Cell proliferating effect of *F. berteroana* ethyl acetate extract (FEAE) and resulting fractions FEAE-F0, FEAE-F1, FEAE-F2 and FEAE-F3 after 24 h and 48 h of treatment of dermal papilla cells with concentrations 0.1 to 50 μg mL^−1^ normalized and compared the control. Error bars represent SEM.

According to Figure 4, the 24-h treatment of minoxidil at 1.25 μg mL^−^^1^ induced a 14% increase in cell growth compared to the control, although non-significant (*p* = *0.06*) using the unpaired Student’s *t*-test. After 48 h, minoxidil, at the three different concentrations tested, showed no positive effect on cells and even lowered cell growth by 10% (*p* = *0.04*) at 12.5 μg mL^−1^ compared to the control (Figure 4). None of the extracts demonstrated a significant effect on the proliferation of DPCs at 0.1 μg mL^−^^1^, after 24 h or 48 h of treatment (Figure 5, Figure 6 and Figure 7, Table S2). At 1.56 μg mL^−1^, CEAE-F5 (Figure 5) and BEAE-F3 (Figure 6) both induced a slight but significant decrease in cell proliferation to 0.92 (*p* = 0.017) and 0.94 (*p* = 0.02) after 48 h of treatment, while FEAE-F2 (Figure 7) increased cell proliferation to 1.14 (*p* = 0.041) after 24 h of treatment.

The 24-h treatment revealed that between 3.13 μg mL^−1^ and 25 μg mL^−1^, all extracts showed either comparable cell viability to that of the control or induced significant cell proliferation. Furthermore, CEAE induced a dose-dependent proliferative effect on DPCs between 3.13 μg mL^−^^1^ and 12.5 μg mL^−1^, with a 20% (*p* = 0.035), 27% (*p* = 0.0004) and 32% (*p* = 0.02) increase at 3.13, 6.25 and 12.5 μg mL^−1^, respectively (Figure 5). This dynamic was seen at higher concentrations for CEWE, FEAE, FEAE-F0 and FEAE-F3 (Figure 7) with a cell proliferation increase ranging between 21% and 25% (0.016 ≤ *p-*value ≤ 0.04) at 25 μg mL^−1^ to an increase between 31% and 50% (0.003 ≤ *p-*value ≤ 0.05) at 50 μg mL^−^^1^. Only CEAE-F3 at 50 μg mL^−1^ was toxic to cells as it caused a 24% decrease (*p* = 0.004) in viability compared to the control, thus dipping below 80% cell viability.

After 48 h of treatment, however, significant cell proliferation was mitigated. The highest cell proliferation value was observed at 12.5 μg mL^−1^ for FEAE-F2 (Figure 7) with a 25% increase (*p* < 0.0001). In Figure 7, at 25 μg mL^−1^, FEAE-related fractions—FEAE-F0, FEAE-F1, FEAE-F2 and FEAE-F3—increased cell growth between 14% and 17% (0.002 ≤ *p-*value ≤ 0.019) compared to the control. At 50 μg mL^−1^, FEAE-F0 and FEAE-F3 induced 18% (*p* = 0.013) and 24% (*p* = 0.003) increases in cell proliferation, respectively, while FEAE-F1 decreased cell viability by 33% (*p* < 0.0001) compared to the control. Conversely, CEAE-F3 in Figure 5 showed a dose-dependent decrease in cell viability starting at 12.5 μg mL^−1^ with 35% (*p* < 0.0001), 57% at 25 μg mL^−1^ (*p* < 0.0001) and 76% (*p* < 0.0001) lower than the control at 50 μg mL^−1^. These results echoed that of CEAE-F3 after 24 h of treatment at 50 μg mL^−1^.

In summary, proliferation-inducing concentrations for FEAE and its fractions FEAE-F0, FEAE-F2 and FEAE-F3 range between 6.25 and 50 μg mL^−1^ for both time points. The optimal range is narrowed down between 6.25 and 25 μg mL^−1^ for FEAE-F1 and mirrors that of *C. inophyllum* extracts CEAE and CEWE and fractions CEAE-F4 and CEAE-F5. BEAE and fractions BEAE-F3 and BEAE-F4 (Figure 6) do not display an optimal range but rather punctual concentrations with proliferative activity.

This led to selecting the extracts and concentrations CEAE-F4, CEAE-F5, BEAE-F4, CEAE, CEWE, BEAE, FEAE, FEAE-F0, FEAE-F1, FEAE-F2, FEAE-F3 and minoxidil at 25, 25, 12.5, 12.5, 25, 12.5, 50, 50, 25, 25, 50 and 1.25 μg mL^−1^ respectively, for RT-qPCR study.

### 2.3. Hair Growth Potential Mediated through Regulation of Hair Growth Factors in DPCs

The relative mRNA expression levels of the 48h treatments versus the control group were calculated for seven genes of interest in cellular mechanisms of hair growth, such as cyclin D1 (*CCND1)*, lymphoid enhancer-binding factor 1 (*LEF1),* dickkopf WNT signaling pathway inhibitor 1 (*DKK1),* Wnt family member 5A *(WNT5A)*, transforming growth factor beta 1 (*TGFΒ1),* peroxisome proliferator activated receptor delta *(PPARD)* and R-spondin 2 (*RSPO2).* RT-qPCR could not be performed on FEAE-F2 because it induced cell death with a lot of debris at 25 μg mL^−1^ when observed under the microscope in the presence of cells in a six-well plate for 48 h.

**Figure 8:**
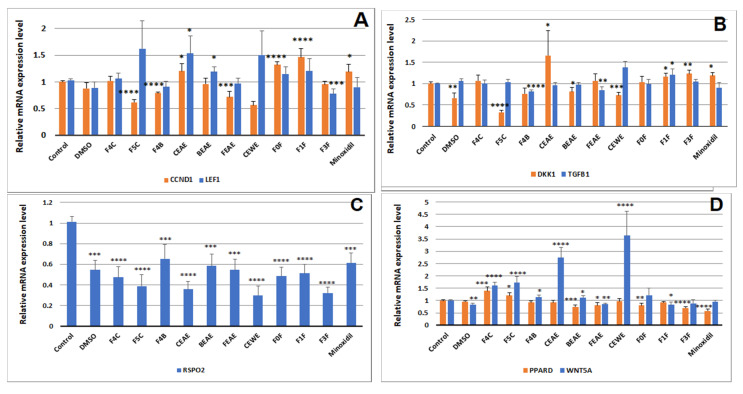
Relative mRNA expression levels of *cyclin D1* and *LEF1* (**A**), *DKK1* and *TGFβ1* (**B**), *RSPO2* (**C**) and *PPARD* and *WNT5A* (**D**) compared to the control. Tested concentrations are 50 μg mL^−1^ for FEAE-F0, FEAE-F3 and FEAE, 25 μg mL^−1^ for CEWE, FEAE-F1, CEAE-F4 and CEAE-F5, 12.5 μg mL^−1^ for BEAE, CEAE and BEAE-F4. Significance is determined by * p < 0.05, ** p < 0.01, *** p < 0.001 and **** p < 0.0001 using a Student’s *t*-test.

*CCND1* is a downstream target gene of the Wnt/β-catenin pathway. During Wnt activation, cytoplasmic β-catenin accumulates and then translocates to the nucleus where it binds to transcription factor Lef(1)/TCF [38,39,40]. The rate of change of the signaling factor stimulates the transcription of several genes, including *CCND1* [41]. In other words, when Wnt is activated, there is an upregulation of *LEF1* and *CCND1* in DPCs. Interestingly, in Figure 8A, CEAE shows a significant increase in the expression of both *CCND1* and *LEF1,* to 1.20 (*p* = 0.03) and 1.53 (*p* = 0.02), respectively. Other extracts increased *CCND1* expression only, such as FEAE-F0, FEAE-F1 and minoxidil to 1.32 (*p* < 0.0001), 1.42 (*p* < 0.0001) and 1.19 (*p* = 0.03), respectively, or only *LEF1,* as BEAE increased to 1.19 (*p* = 0.02). It is noteworthy that CEAE-F5 and CEWE demonstrate the strongest yet non-significant increase in *LEF1* to 1.63 and 1.50, respectively.

Additionally, both TGFB1 and DKK1 have been shown to be paracrine factors in DPCs that are expressed during late anagen phase to suppress keratinocyte development and thus contribute to the anagen-catagen phase transition [42,43,44]. Indeed, DKK1 protein negatively regulates the canonical Wnt pathway. It binds to the receptor LRP5/6, blocking Wnt ligands and causing a halt in downstream signaling in both keratinocytes and DPCs [44,45]. A previous study showed that its gene expression matches that of the protein [44], so studying the downregulation of *DKK1* and *TGFB1* by our extracts is a strong indicator of the protein levels. Upon first calculations, the expression level of *DKK1* is downregulated by CEAE-F5 three-fold to 0.33 (*p* < 0.0001) and one-and-a-half-fold to 0.66 (*p* = 0.003) by DMSO, as well as to 0.76 (*p* = 0.04), 0.81 (*p* = 0.03) and 0.73 (*p* = 0.0007) by BEAE-F4, BEAE and CEWE (Figure 8B). Nevertheless, DMSO is our solvent control. To eliminate the potential effect of DMSO on the extracts, the relative expression was calculated compared to DMSO. Only CEAE-F5 significantly decreased *DKK1* expression two-fold (*p* = 0.02) (Table S3). As for *TGFB1,* its mRNA expression level was significantly decreased by BEAE-F4 and FEAE to 0.81 (*p* < 0.0001) and 0.85 (*p* = 0.004), respectively (Figure 8B).

*PPARD* is upregulated to 1.40 (*p* = 0.0005) and to 1.21 (*p* = 0.02) by CEAE-F4 and CEAE-F5, respectively (Figure 8D). It is a downstream target gene of the Wnt pathway, leading to an increase in mRNA expression when Wnt/β-catenin is most active, during the anagen phase. R-spondins, including R-spondin 2 (RSPO2) are proteins that interact in the activation of the Wnt pathway and they are expressed by DP cells at the onset of the anagen phase [23,30]. Similarly to *DKK1*, DMSO has a significant decreasing effect on *RSPO2* expression that could be responsible for the downregulation observed in extracts (Figure 8C). Upon re-normalizing relative gene expression to that of DMSO, none of the extracts showed a significant effect on the gene levels (Table S3). Perhaps studying *RSPO2* levels in other cell types would give a more distinct impression of its contribution to hair follicle growth [46].

While the WNT5Aprotein, a non-canonical Wnt ligand, is believed to attenuate the canonical Wnt pathway in DPCs [26], its gene expression is strongest during the anagen phase. The mRNA level of *WNT5A* is upregulated by CEAE-F4, CEAE-F5, BEAE-F4, CEAE, BEAE and CEWE to 1.61 (*p* < 0.0001), 1.73 (*p* < 0.0001), 1.14 (*p* = 0.04), 2.74 (*p* < 0.0001), 1.13 (*p* = 0.03) and 3.64 (*p* < 0.0001), respectively.

## 3. Discussion

Hair loss is an increasingly prevalent phenomenon occurring in both men and women. Our study aimed to determine the hair growth-inducing activity of three plants of the Polynesian cosmetopoeia, *B. pilosa*, *C. inophyllum* and *F. berteroana*. We have shown that our extracts demonstrate their hair growth activity by increasing proliferation of dermal papilla cells. They also seem to target the Wnt/β-catenin pathway as well as the TGFβ pathway. Also, their chemical compositions reveal potential bioactive compounds. Indeed, the results of the cell proliferation assay on DPCs showed that the ethyl acetate extract of *F. berteroana*, FEAE, and fractions FEAE-F0, FEAE-F1, FEAE-F2 and FEAE-F3, exhibit strong proliferative activity on DPCs between 6.25 and 25 μg mL^−1^ after 24h and 48h of treatment with increases of up to 25% compared to the control. *C. inophyllum* extracts CEAE and CEWE and fractions CEAE-F4 and CEAE-F5 increase cell proliferation in the dermal papilla between 6.25 and 25 μg mL^−1^ after 24 h. These findings are similar to several extracts reported in the literature for stimulating hair growth via proliferation of DP cells such as the marine alga *Ishige sinicola* ethanol extract that increased cell proliferation by 5.5% compared to the control at 10 μg mL^−1^ after 4 days of treatment [47] or a methanol extract of *Geranium sibiricum* that induced a 32.7% increase in DPCs after 24 h of treatment [48]. Senescent DP cells progressively undergo apoptosis and lose their proliferative activity, leading to gradual hair thinning [49]. Furthermore, a decline in the pool of DPCs per hair shaft leads to shorter and thinner hair [21]. Hence, keeping an active pool of dermal papilla cells throughout the hair cycle is paramount for length retention and hair growth. This suggests that our extracts mediate their hair growth activity by stimulating proliferation of the dermal papilla cells.

It is important to note that the mesenchymal-epithelial crosstalk lies at the root of hair follicle growth [50,51]. The dermal papilla serves as a physical niche for progenitor cells as well as a signaling center for their proliferation and differentiation into epithelial cells, such as keratinocytes that form the hair follicle [23]. The DP cell signature genes and how its signaling evolves during the hair cycle have been studied to assess its cues to surrounding epithelial and bulge stem cells [51,52,53]. Indeed, a disruption in these cues causes gradual hair loss [40,54]. Previous studies have shown that TGFβ1 and 2 of the TGFβ pathway are involved in androgen-induced androgenetic alopecia (AGA) by promoting catagen phase entry [43]. TGFβ1 and 2 induced recruitment of caspases 3 and 9 and caused cell apoptosis [55]. In contrast, it was discovered that TGFβ inhibition by antibody antagonists suppressed entry into the catagen phase [55]. Additionally, a TGFβ1 inhibitor, TP0427736, inhibited TGFβ1-induced phosphorylation of Smad proteins 2/3 as well as elongated the anagen phase in mouse hair follicles [56]. Upon RT-qPCR analysis, FEAE and BEAE-F4 decreased *TGFB1.* This could imply a hair growth-promoting activity potentially via attenuation of the TGFβ pathway. According to the previously mentioned studies, inhibition of its signaling would allow entry into a new anagen phase as Wnt signaling overpowers TGFβ signaling, thus preventing epithelial cell apoptosis. This can be further understood because even the mRNA levels of TGFβ receptors I and II are upregulated in balding hair follicles, alongside the increase in TGFβ1 protein levels [57]. Conversely, AGA is also caused by a simultaneous attenuation of the Wnt/β-catenin pathway in balding hair follicles. Furthermore, β-catenin activity in balding DP cells is significantly reduced compared to non-balding DP cells [57]. This further stresses the importance of canonical Wnt signaling in hair growth regulation. Our extract CEAE-F5 seems to target the Wnt/β catenin pathway. The observed DPC proliferation is hypothesized to be a result of the downregulation of *DKK1*, one of the pathways’ inhibitors, which allows the increase in Wnt signaling, as demonstrated by upregulated levels of *PPARD* and *LEF1*. VB-1, a compound shown to exert hair growth-promoting effects, also caused an increase in the expression levels of *LEF1* and *WNT5A*, as well as BPMP2 and *BMP4*, concomitant with a decrease in *DKK1*, *AXIN2* and *TGFB1* levels, amongst others [58]. As for CEAE, according to the studied genes, its specific target remains uncertain, but the canonical Wnt pathway also seems to be involved, as suggested by *CCND1* and *LEF1* upregulation.

We studied the chemical composition of the phytoextracts and fractions both through colorimetric assays and UPLC-MS/MS to gain better knowledge of the potential bioactive compounds present as well as assess their general chemical diversity. All the tested extracts contained the studied families, i.e., polyphenols, flavonoids, anthocyanins and saponins. The UPLC-MS/MS analysis also enabled the tentative identification of an array of compounds. Flavonoids and derivatives (*O*-glycosyl and flavan-3-ols), the main molecules identified in our extracts, are believed to potentiate hair growth by promoting vascularization near cells via vascular endothelial growth factor VEGF and its receptor VEGFR [59,60] notably naringenin, quite similarly to minoxidil for DPCs. Furthermore, procyanidin B2 was shown to promote hair follicle growth and its action was attributed to the inhibition of protein kinases C α, βI, βII and η [61]. Reported studies showed that activation of these protein kinases inhibits hair growth [62,63] as well as hair pigmentation [64]. We identified procyanidin B2 in *C. inophyllum* extracts, which gives rise to other potential targets to consider for further study of the hair growth activity of the extracts. Sinapic acid [65] and 3,4,5-tri-*O*-caffeoylquinic acid [66] both promote hair growth via several factors, namely stimulation of β-catenin production. Coumaric and sinapic acids are both hydroxycinnamic acids, and coumaric acid was identified in all three ethyl acetate extracts of *F. berteroana, C. inophyllum* and *B. pilosa* (Table 1), further pointing towards a potential Wnt/β-catenin target stimulation of hair growth of our extracts and fractions. Several other natural compounds have shown hair growth activity such as a tannin, corilagin [48,61], chalcones with 3-deoxysappanchalcone [67], terpenes, such as costunolide [68], or ginsenoside Rb1 [69,70]. A derivative of benzodiazepine named tianeptine was also found to have an effect on hair growth [45]. Other compounds extracted from both plant and marine species [47,71] were reported to have hair-stimulating activities. Further dereplication study of known compounds’ corresponding peaks in our LC-MS/MS analyses, as well as targeted isolation and identification of novel molecules in our extracts, will help to elucidate potential bioactive compounds and their molecular targets.

Overall, this study has shown that CEAE-F5 and CEAE, as well as BEAE-F4 and FEAE, mediate their hair growth activity by proliferation of DPCs. Additionally, the first two potentially upregulate Wnt/β-catenin signaling, while BEAE-F4 and FEAE could mitigate the TGFβ pathway, although these findings require further investigation for confirmation. In addition, the UPLC-MS analysis of the extracts and fractions leads us to believe that upon isolation, several compounds from the extracts could be vasodilators and increase blood and nutrient flow to DPCs, as they contain many flavonoids. These results support our hypothesis concerning hair growth activity via DP cell proliferation. They also tentatively suggest potential gene targets of our extracts. To our knowledge, this is the first time that *F. berteroana* has been studied for its hair growth activity. Nevertheless, these findings remain tentative and only partly explain the extracts’ hair growth effect, as many factors come in to play to promote healthy hair growth or prevent hair loss. Further study considering other aspects of hair loss prevention will be necessary to confirm both our biological and chemical results and go deeper into the broader mechanisms employed by the extracts to exert their hair growth activity.

## 4. Materials and Methods

### 4.1. Plant Material and Extraction

*Bidens pilosa* L. whole plant, *Calophyllum inophyllum* L. leaves and *Fagraea berteroana* A.Gray ex Benth. fruits were collected between December 2017 and April 2018 in Tahiti, French Polynesia. They were identified by the botanist Jean-François Butaud and vouchers (JF BUTAUD & K HUGHES 3594; K HUGHES 8 and K HUGHES 4) were deposited at the herbarium of French Polynesia (PAP) (see previous publication Hughes et al. 2019). The different parts were oven-dried at 40 °C for 2 days then ground to a 2 mm powder. The powders obtained were macerated in ethyl acetate, ethanol: water (50:50) or water for 12 h, under agitation. Ethyl acetate was evaporated until a solid extract was obtained. An aliquot of each dried extract was later dissolved in dimethyl sulfoxide to obtain a solution used for biochemical screenings. The aqueous ethanol (CEWE) and aqueous extracts (CWE) of *C. inophyllum* were evaporated then freeze-dried for complete drying and dissolved in dimethyl sulfoxide.

After thin layer chromatography (TLC) analysis of resulting extracts, *B. pilosa* and *C. inophyllum* ethyl acetate extracts (BEAE and CEAE) were fractionated using the Combiflash Companion Teledyne Isco (Teledyne ISCO, Lincoln, NE, USA) along with a 220 g RediSep Rf Teledyne Isco column (Teledyne ISCO, Lincoln, NE, USA) in normal phase with silica gel. The flow rate was set at 100 mL min^−1^ with ethyl acetate (**A**) and cyclohexane (**B**). The elution program consisted of a gradient solvent system from 80% to 50% solvent **B** for 5 min, followed by 8 min of 50% **B** in isocratic mode, then 8 min of 30% **B**. This was followed by 8 min of 20% **B**, then 15 min of 100% **A** and ended with 10 min in 100% methanol.

This method generated approximately 200 fraction tubes each for BEAE and CEAE. The subfractions were combined into main fractions through TLC and HPLC analysis and control. BEAE yielded four main fractions, BEAE-F1, BEAE-F2, BEAE-F3, and BEAE-F4. CEAE yielded five main fractions, CEAE-F1, CEAE-F2, CEAE-F3, CEAE-F4 and CEAE-F5.

The ethyl acetate *Fagraea berteroana* extract (FEAE) was fractioned (10.89 g) using open column chromatography with silica gel 60 Å.

The solvents used for gradient elution were cyclohexane, ethyl acetate and methanol in a gradually polar mixture of solvents. The elution started with two column volumes of cyclohexane/EtOAc (80/20) followed by one column volume of cyclohexane/EtOAc (60/40) which lead to the first fraction, FEAE-F0. The second fraction, FEAE-F1, was obtained by recovering and evaporating the 2nd volume of cyclohexane/EtOAc (60/40) and the first volume of cyclohexane/EtOAc (70/30). FEAE-F2 was composed of the 2nd volume of cyclohexane/EtOAc (70/30) and 100% EtOAc, and finally FEAE-F3 was the fraction eluted with 100% methanol.

### 4.2. Determination of Polyphenol, Flavonoid, Anthocyanin and Saponin Contents

The polyphenolic content was determined using a colorimetric method. A standard curve was plotted by preparing different concentrations of gallic acid solutions and measuring their absorbance in the presence of phosphotungstic acid and 15% carbonate sodium at 710 nm. The polyphenol content of the extracts was obtained from the standard curve and expressed as milligrams of gallic acid equivalents per g of dry extract. Each measure was taken in triplicate.

The flavonoid content was determined according to a slightly modified assay [72] by plotting a standard curve of rutin solutions at different concentrations and measuring their absorbance at 425 nm in the presence of 2% aluminum chloride after 15 min of incubation. The absorbance of the blank measure for each extract—without added aluminum chloride—was subtracted to the value of the measure in the presence of the reagent. Flavonoid content was calculated from the standard curve and expressed as milligrams of rutin equivalents per g of dry extract.

The anthocyanin content of the extracts was determined by preparing solutions of kuromanin at different concentrations. The absorbance of the solutions in the presence of 0.1% hydrochloric acid was measured at 524 nm to plot the standard curve. Anthocyanins were calculated through the standard curve and expressed as milligrams of kuromanin equivalents per g of dry extract.

The saponin content [73] was determined by plotting a standard curve of different concentrations of jujuboside A solutions. Ethanolic vanillin at 8% and 72% sulfuric acid were added to the solutions and left to incubate for 10 min exactly at 60 °C. The reaction was stopped by putting the tubes into ice-cold water. The absorbance was then measured at 544 nm. The saponin contents of the extracts were determined with the standard curve and expressed as centigrams of jujuboside A equivalents per g of dry extract.

### 4.3. UHPLC-MS/MS Analysis

The chemical analyses were performed on a UHPLC system (Dionex Ultimate 3000, Thermo Scientific^®^ equipped with a photodiode array detector: 254, 280, 340 and 450 nm) coupled to a high-resolution mass spectrometer (HRMS QqToF Impact II equipped with an electrospray ionization source, Bruker Daltonics, Germany) in positive and negative ionization modes (20 eV and 40 eV). The extracts were prepared by solubilizing 1 mg of dry extract in 1 mL of methanol then filtered with a 0.2 µm syringe filter. The separations were carried out on an Acclaim RSLC C18 column (2.1 mm × 150 mm, 2.2 µm, DIONEX, Sunnyvale, CA, USA) at 40 °C by injecting 1 µL of the prepared solution. A smaller volume of extracts (0.25 µL and 0.5 µL) was injected when extracts were too concentrated. Additionally, some extracts were diluted 50 times to avoid detector saturation.

Two chromatographic analytical methods, depending on the polarity of the extracts, were developed to obtain the best chromatographic separation, with H_2_O + 0.1% formic acid (solvent **A**) and acetonitrile + 0.1% formic acid (solvent **B**). The first program (pg 1) was as described: 2 min at 5% **B**, then 7 min ranging from 5 to 50% **B** followed by 2 min at 50% in isocratic mode. Finally, a 2-min isocratic wash at 100% B and a re-equilibration step at 5% for 3 min ended the analytical program (flow rate at 0.5 mL min^−1^). It was performed on extracts BEAE-F3, BEAE-F4, CEAE-F2, CEAE-F3, CEAE-F4, CEAE-F5, FEAE-F1, FEAE-F2 and FEAE-F3. The 2nd program (pg 2) consisted of 2 min at 5% **B** followed by a linear gradient up to 100% B for 8 min, then 100% **B** for 3 min and ended by a 3 min re-equilibration at 5% **B** (same flow rate). This second method was used for the less polar extracts, BEAE-F1, BEAE-F2, CEAE-F1 and FEAE-F0. The injection of a formate acetate solution in basic media forming clusters on the studied mass range was used for mass calibration before each analysis.

Mass spectra were acquired ranging from 50 to 1200 *m*/*z* at 2Hz. The nebulizer pressure was set at 50.8 psi, the capillary)) voltage at 3000 V, the dry gas flow rate at 12 L.min^−1^ and dry temperature at 200 °C.

The MS/MS spectral data obtained were compared to entries in repositories such as MassBank of North America (MoNA) and MassBank, the METLin library and/or literature data when available. The large number of extracts and fractions led to focusing on the spectra obtained for the extracts. The main compounds present in all samples (extracts and fractions) were presented, as well as several significant peaks, specific to a species to demonstrate the variety of compounds present in the samples.

### 4.4. Ferric Reducing Antioxidant Power (FRAP) Assay

A volume of 50 μL of the tested extract was mixed with 50 μL of distilled water in a 96-well Greiner plate. A volume of 200 μL of FRAP solution was then added and the plate was left to incubate for an hour at 37 °C to evaluate the antioxidant properties of the extracts. The resulting absorbance was read at 593 nm with a SPARK® multimode microplate reader (TECAN, Switzerland) [74,75].

A standard curve was plotted by preparing different concentrations of Trolox ranging from 1 to 20 mg L^−1^.

O.D. = a × [Trolox] + b(1)

The antioxidant activity of the extracts was determined in μmol Trolox equivalent g^−1^ of dry matter. All results were obtained in triplicate and the standard deviation (SD) was calculated for each value.

### 4.5. 5-Lipoxygenase (5-LOX) Assay

Each sample was tested at different dilutions in a quartz cuvette to assess the anti-inflammatory activity of the extracts. The enzymatic control was obtained by a volume of 2.95 mL of phosphate buffer at pH = 9 mixed with 30 μL of the sample (or 10 μL of standard + 20 μL of buffer), 10 μL of linoleic acid and 10 μL of 5-lipoxygenase at 50,000 U mL^−1^. Nordihydroguaiaretic acid (NDGA) was used as positive control where a volume of 2.97 mL of buffer was mixed with 10 μL of the standard at different concentrations, 10 μL of linoleic acid and 10 μL of 5-lipoxygenase at 50,000 U mL^−1^. The blank solutions of the samples and standard were obtained by replacing linoleic acid with phosphate buffer. The absorbance was read at 233 nm for 60 s with 10 s intervals on a U-2001 Hitachi spectrophotometer [76]. For each concentration, the 5-LOX inhibition percentage was determined as follows Equations (2) and (3):

% inhibition (at a given concentration) = 100 (([O.D. sample]_t = 1 min_ − [O.D. sample]_t = 0 min_) × 100/([O.D. control]_t = 1 min_ − [O.D. control]_t = 0 min_)). (2)

(3) $$\begin{array}{l} \%\;\mathrm{inhibition}\;(\mathrm{at}\;a\;\mathrm{given}\;\mathrm{concentration})\\ \;=100\;\frac{\left({\left[O.D.\;\mathrm{sample}\right]}_{t=1\;\min}\;-\;{\left[O.D.\;\mathrm{sample}\right]}_{t=0\;\min}\right)\;\times \;100}{\left({\left[O.D.\;\mathrm{control}\right]}_{t=1\;\min}\;-\;{\left[O.D.\;\mathrm{control}\right]}_{t=0\;\min}\right)} \end{array}$$

The IC50 value was calculated by plotting an inhibition curve between the inhibition percentages and sample concentrations.

### 4.6. Primary Culture of Human Hair Follicle Dermal Papilla Cells (HFDPCs)

Human hair follicle dermal papilla cells (HFDPCs) were purchased from Promocell (Heidelberg, Germany) and grown in follicle dermal papilla cell growth medium (Promocell, Sickingenstr, Heidelberg, Germany) supplemented with 100 U mL^−1^ penicillin and 100 μg mL^−1^ streptomycin (Gibco). The cells were cultured in a humidified atmosphere at 37 °C and 5% CO_2_.

### 4.7. Measurement of Cell Viability with the MTT Assay

The Cell Proliferation kit I MTT assay (Roche, Manheim, Germany) was performed to assess the influence of the extracts on the viability of HFDPCs. Cells were seeded (10^4^ and 8 × 10^3^ for 24- and 48-h experiments, respectively) into 96-well plates. They were treated with 200 μL of increasing concentrations of the extracts, ranging from 0.1 μg mL^−1^ to 50 μg mL^−1^. The control group consisted of DMSO diluted in medium to concentrations ranging from 0.001% to 0.5%, similarly to those of the extracts. The plates were incubated at 37 °C and 5% CO_2_ for 24 h and 48 h independently. The supernatant was then discarded and 100 μL of 10% MTT in fresh medium was added to the wells and incubated for 4 h before adding 100 μL of solubilization solution. The plates were incubated overnight, and the absorbance was read on a Multiskan GO spectrophotometer (Thermoscientific, Inc., Vantaa, Finland) at 570 and 690 nm. All tests were done at least in triplicate. The absorbance of each extract concentration was normalized to its corresponding control. Hence, all control values are equal to 1 ± SEM. For visual clarity, in Figure 5, Figure 6 and Figure 7, only one control was plotted and the error bars added correspond to those of the 24 h treatment.

### 4.8. RNA Isolation, Reverse Transcription and Quantitative Real-Time Polymerase Chain Reaction (qRT-PCR)

Total RNA was extracted from cells using TRIzol^®^ reagent (Thermo Fisher Scientific, Inc., Carlsbad, CA, USA). After quantification on a Nanodrop 1000 spectrophotometer (ThermoFisher Scientific, Inc., Wilmington, DE, USA), 750 ng of RNA were reversed transcribed to cDNA with Superscript IV (ThermoFisher Scientific, Inc., Vilnius, Lithuania). The qRT-PCR was performed to detect the expression of genes of interest using SYBR-Green PCR Master Mix (Roche, Mannheim, Germany) with a Lightcycler 480 II Roche system (Roche, Manheim, Germany). The thermocycling conditions were as follows: initial denaturation at 95 °C for 2 min, then denaturation at 95 °C for 15 s, annealing at 56 °C for 15 s and extension at 68 °C for 20 s, for a total of 40 cycles. The gene expression was normalized to CALM2, GAPDH and PUM1 expressions. The expression level of the genes of interest was calculated using the 2^−ΔΔCq^ method. Primers were designed and obtained from Eurogentec (Table 3). The expression level obtained after the 2^−ΔΔCq^ of each treatment for a given gene was finally normalized to the replicate mean of its corresponding control. Expression level (4) graphs in Figure 8 represent fold changes induced by extracts compared to the control.

(4) $$Expression\;level=\frac{2^{-∆∆Cq}\left(extract\;for\;gene\;x\right)}{average\left(2^{-∆∆Cq}\;control\;for\;gene\;x\right)}$$

The expression change was calculated as the difference between the control (1 or 100%) and the treatment with extracts.

### 4.9. Statistical Analysis

Data on cell-based tests were expressed as mean ± SE (standard error) of at least three independent experiments. The statistical tests were performed on Stata14. An *F*-test was performed to determine equality of the variances between the control group mean and each treatment group mean, followed by an unpaired Student’s *t*-test. Welch’s *t*-test was performed if variances were unequal. A *p*-value ≤ 0.05 was considered significant.

## 5. Conclusions

CEAE, CEAE-F5, BEAE-F4 and FEAE from plants *B. pilosa, C. inophyllum* and *F. berteroana* showed interesting hair growth properties by inducing cell proliferation of HFDPCs even at concentrations near 6 μg mL^−1^. Furthermore, the UPLC-MS analysis of the extracts revealed several compounds, flavonoids, tannins and anthocyanins, that are known to have hair proliferative activities which could explain their traditional use in hair treatment. Extracts CEAE and CEAE-F5 are believed to partly mediate their activity via the *Wnt/β* catenin pathway through the modulation of several genes of factors involved in that pathway. The BEAE-F4 and FEAE results suggest that they might mediate their hair growth activity via a pathway that was not extensively studied here, the TGFβ pathway.

## Figures and Tables

**Table 1:** LC-MS/MS details of compounds detected in extracts and analyzed at 40 eV in negative ion mode.

#	Experimental Ion Mass (*m/z*)	Molecular Formula (Error in ppm)	Tentative Identification	Presence	Comparison Source
**1**	153.0195	C_7_H_6_O_4_ (0.5)	dihydroxybenzoic acid	CEWE, BPLA (adduct)	PR100601 (MassBank)
**2**	173.0455	C_7_H_10_O_5_ (0.1)	Shikimic acid	CEAE, CEWE	RP017513 (MassBank)
**3**	163.0402	C_9_H_8_O_3_ (−0.7)	2-coumaric acid	BEAE, CEAE, FEAE	KO000444 (MassBank)
**4**	301.0354	C_15_H_10_O_7_ (−1.2)	quercetin	ALL	PB002412 (MassBank)
**5**	359.0771	C_18_H_16_O_8_ (0.3)	trihydroxy-trimethoxyflavone	BEAE	
**6**	375.1303	C_16_H_24_O_10_ (−1.6)	loganic acid	FEAE, FEFE	KO001304 (MoNA) VF-NPL−QEHF012680 (MoNA)
**7**	433.1140	C_21_H_22_O_10_ (0.1)	prunin (naringenin 7-*O*-glucoside)	CEAE, CEWE	E.I.T.
**8**	447.0933	C_21_H_20_O_11_ (−0.2)	luteolin-4′-*O*-glucoside	BEAE	PR100805 (MassBank)
**9**	447.0933	C_21_H_20_O_11_ (−0.4)	quercitrin (quercetin-3-rhamnoside)	ALL	FIO00587, FIO00586, BS003205 (MoNA)
**10**	447.0933	C_21_H_20_O_11_ (−1.3)	kaempferol-7-glucoside	BEAE, CEAE, CEWE, CWE	Yamagaki et al., 2014 [34]
**11**	461.1090	C_22_H_22_O_11_ (−0.2)	scutellarein-7-glucuronide	CEAE, CEWE	BS003571 (MassBank)
**12**	463.1240	C_22_H_24_O_11_ (1.1)	viscumiside A	BEAE	CCMSLIB0000008176 (MoNA)
**13**	477.0669	C_17_H_18_O_13_ (0.3)	quercetin-3-*O*-glucuronide	BEAE	VF-NPL-QTOF009454 (MoNA) RIKENPlaSMA006716 (MoNA)
**14**	489.1414	C_24_H_26_O_11_ (−2.4)	trimethylisoorientin	CEAE, CEWE, CWE	E.I.T., PR040130 (MoNA)
**15**	511.0554	C_21_H_20_O_13_S (−1.4)	kaempferol-C_6_H_10_O_7_S	CEWE, CWE	E.I.T.
**16**	527.0500	C_21_H_20_O_14_S (−1.1)	quercetin-C_6_H_10_O_7_S	CEWE, CWE	E.I.T.
**17**	577.1341	C_30_H_26_O_12_ (1.9)	procyanidin B2	CEAE, CEWE, CWE	BS003942 (MassBank)
**18**	579.1360	C_26_H_28_O_15_ (−0.8)	kaempferol-3-hexoside-pentoside	CEAE, CEWE, CWE	E.I.T.

E.I.T. = Explanation in text. # = number

**Table 2:** Antioxidant potential (FRAP) and anti-inflammatory (5-LOX) activity of the extracts and fractions of the three species.

Species	Extracts/Fractions	FRAP Assay		5-LOX	
		Trolox Equivalent (μmol.g^−1^ of Dry Matter)	SD	IC_50_ (μg mL^−1^)	Inhibition (% of 5-LOX)
*Calophyllum inophyllum*	CWE	992	24	55	50
	CEAE	1328	110	88	39
	CEAE-F1	247	19	61	38
	CEAE-F2	415	6	28	50
	CEAE-F3	910	15	20	50
	CEAE-F4	909	28	20	50
	CEAE-F5	2192	66	21	50
*Bidens pilosa*	BEAE	1383	103	81	50
	BEAE-F1	192	12	31	50
	BEAE-F2	536	9	11	8
	BEAE-F3	1262	20	20	6
	BEAE-F4	3141	129	57	50
*Fagraea berteroana*	FEAE	632	49	137	50
	FEAE-F0	42	2	-	-
	FEAE-F1	59	3	33	9
	FEAE-F2	175	6	94	19
	FEAE-F3	289	33	74	50
	Green tea	14947	868		
	NDGA			1	50

*Calophyllum inophyllum* aqueous extract = CWE, ethyl acetate extract = CEAE. *Bidens pilosa* ethyl acetate extract = BEAE and *Fagraea berteroana* ethyl acetate extract = FEAE. NDGA = nordihydroguaiaretic acid.

**Table 3:** List of designed primers sequences used for qRT-PCR analysis.

Gene Names	Forward Primer	Reverse Primer
*CALM2*	5′-GGG-AAC-ATC-TGG-GTT-ATG-CC-3′	5′-GAC-TGT-CCA-TAG-TCC-ACG-CA-3′
*CCND1*	5′-AAC-TAC-CTG-GAC-CGC-TTC-CT-3′	5′-CCA-CTT-GAG-CTT-GTT-CAC-CA-3′
*DKK1*	5′-TCC-GAG-GAG-AAA-TTG-AGG-AA-3′	5′-CCT-GAG-GCA-CAG-TCT-GAT-GA-3′
*GAPDH*	5′-CCA-GCA-AGA-GCA-CAA-GAG-GA-3′	5′-TGG-TTG-AGC-ACA-GGG-TAC-TT-3′
*LEF1*	5′-GCT-GCC-TAC-ATC-TGA-AAC-ATG-G-3′	5′-GGA-TCA-GCG-TCT-CTA-GCA-GT-3′
*PPARD*	5′-TTC-CAG-CAG-CTA-CAC-AGA-CC-3′	5′-TGA-ACA-CCG-TAG-TGG-AAG-CC-3′
*PUM1*	5′-GGT-GCC-CTT-GTA-GTG-AAT-GC-3′	5′-TGT-TGT-TCC-AGC-AAG-ACC-AC-3′
*RSPO2*	5′-CAG-CCT-CAC-ACC-TCT-AGC-AT-3′	5′-CTG-CTC-TGC-CCA-GTA-TCT-GT-3′
*TGFB1*	5′-CTG-GCG-ATA-CCT-CAG-CAA-CC-3′	5′-CGG-TAG-TGA-ACC-CGT-TGA-TGT-C-3′
*WNT5A*	5′-GAG-AGT-GCT-CGC-ATC-CTC-AT-3′	5′-GCC-ACA-TCA-GCC-AGG-TTG-TA-3′

## Supplementary Materials

The following are available online. Table S1: Detailed MS^2^ data, Table S2: DPC proliferation values-SEM-*p*-values, Table S3: RT-qPCR Gene expression comparison.
